# Supplementary material for: Stability of ZIF-8 Nanoparticles in Most Common Cell Culture Media
Source: Molecules. 2022 May 18;27(10):3240. doi: 10.3390/molecules27103240 (PMC9144353; doi:10.3390/molecules27103240)
Supplement: Supplementary file 1 [file molecules-27-03240-s001.zip › molecules-1720025-supplementary.pdf]

SUPPORTING INFORMATION

Stability of ZIF-8 nanoparticles in most common cell culture media

Anna S. Spitsyna[1,2], Artem S. Poryvaev,[1] Natalya E. Sannikova[1], Anastasiya A. Yazikova,[1] Igor A. Kirilyuk,[2] Sergey A. Dobrynin,[2] Olga A. Chinak,[3] Matvey V. Fedin [1*] and Olesya A. Krumkacheva[1*]

[1] International Tomography Center SB RAS, Novosibirsk, 630090, Russia
[2] N.N.Vorozhtsov Institute of Organic Chemistry SB RAS Novosibirsk, 630090, Russia
[3] Institute of Chemical Biology and Fundamental Medicine, Novosibirsk, 630090, Russia

Table of contents

Cell culture media composition 2

Results of simulation of experimental spectra. 4

Parameters β, Ka, and fraction of spin probes escaped from ZIF-8 for studied amino acids. 5

Particle size distribution 6

CW EPR spectra of initial R@ZIF-8 and R@ZIF-8 5-fold diluted with water 7

CW EPR of R@ZIF-8 in different media 7

Spectra of R@ZIF-8 in MEM at different concentrations 8

EPR spectra of R@ZIF-8 with amino acids 9

EPR spectra at different concentrations of amino acids 9

EPR spectra of samples with 2-methylimidazole (MIM) addition. 10

Cytotoxic activity of MIM in A549 cells. 11

Cell culture media composition

Table S1. Cell culture media composition. The concentration units of all compounds are 10^{-5} M. Color code for media names corresponds to the colors in Figure 3.

	MEM	199	RPMI	alpha-MEM	DMEM	IMDM	L15
**L-Alanine**	0	28	0	28	0	28	250
**L-Arginine**	60	33	110	60	40	40	290
**L-Asparagine**	0	30	170	33	0	19	7,6
**Aspartic acid**	0	23	15	23	0	23	0
**L-Cysteine**	0	0,056	0	57	0	0	99
**L-Cystine**	10	9,9	21	10	20	29	0
**L-Glutamate**	0	45	14	51	0	51	0
**L-Glutamine**	200	68	210	200	400	400	210
**L-Glycine**	0	67	13	67	40	40	27
**L-Histidine**	20	10	9,7	20	20	20	160
**Hydroxyproline**	0	7,6	15	0	0	0	0
**L-Isoleucine**	40	15	38	40	80	80	95
**L-Leucine**	40	46	38	40	80	80	95
**L-Lysine HCl**	40	38	22	40	80	80	51
**L-Methionine**	10	10	10	10	20	20	50
**L-Phenylalanine**	20	15	9,1	19	40	40	76
**L-Proline**	0	35	17	35	0	35	0
**L-Serine**	0	24	29	24	40	40	190
**L-Threonine**	40	25	17	40	80	80	250
**L-Tryptophan**	4,9	4,9	2,5	4,9	7,8	8	9,8
**L-Tyrosine**	20	22	11	23	40	39	170
**L-Valine**	40	21	17	39	80	80	85
**Total aminoacids concentration**	**545**	**577**	**788**	**864**	**1068**	**1232**	**2115**

	MEM	199	RPMI	alpha-MEM	DMEM	IMDM	L15
Phenol Red•Na	2,7	4,5	1,3	2,9	4	4,239871	2,7
CaCl2	180	130		180	180	149	130
KCl	530	530	530	53	530	443	530
KH2PO4		44					44
MgCl2				2000			
MgSO4	81	81	40	81	81	81	160
NaCl	12000	14000	10000		11000	7709	14000
NaHCO3	2600		420	2600	4400	3600	
NaH2PO4	100				91	91	
Na2HPO4		40	560				0
D-Glucose	560	560	1100	560	2500	2498	
D-Galactose							5000

	MEM	199	RPMI	alpha-MEM	DMEM	IMDM	L15
Pyruvic Acid•Na				100	100	99	500
Linoleic acid					0,03		
Lipoic acid				0,097			
Para-aminobenzoic acid		0,036	0,73				
L-ascorbic acid		0,028		25			
Biotin		0,0041	0,082	0,041		0,005	
Choline Chloride	0,71	0,36	2,1	0,71	2,9	2,86	0,71
Folic Acid	0,23	0,0023	0,23	0,23	0,91	0,91	0,23
Myo-inositol	1,1	0,028	19	1,1	4	4,00	1,1
Nicotinamide	0,82	0,02	0,82	0,82	3,3	3,28	0,82
Nicotinic acid		0,02					
DL-Pantothenic Acid (hemicalcium)	0,42	0,0042	0,11	0,42	1,7	1,67	0,42
Pyridoxine•HCl		0,012	0,49				
Pyridoxal •HCl	0,49	0,012		0,49	2	1,96	
Riboflavin	0,027	0,0027	0,053	0,027	0,11	0,11	0,0019
Thiamine	0,3	0,003	0,3	0,3	1,2	1,33	0,24
α-Tocopherol		0,0023					
B12			0,00037	0,1		0,001	
Hypoxanthin		0,22					
Fe(NO3)3 9H2O					0,025		
FeSO4 7H2O							
ZnSO4 7H2O							
CuSO4 5H2O							
Thymidine				4,1			
Adenine SO4		5,4					
Adenosine				3,7			
AMP		0,058					
ATP		1,8					
Cytidine				4,1			
D-adenosine				4			
D-cytidine				4,2			
D-guanosine				3,7			
2-deoxyribose		0,37					
Guanine		0,16					
Guanosine				3,5			
D-ribose		0,33					
Thymine		0,24					
Uracil		0,27					
Uridine				4,1			
Xanthine		0,2					
Tween-80		1,8					
Putrescine							

	MEM	199	RPMI	alpha-MEM	DMEM	IMDM	L15
Calciferol		0,025					
P-retinol acetate		0,035					
HEPES						2500	
Sodium acetate		0,45					
Na2SeO3						0,01	
KNO3						0,08	

Results of simulation of experimental spectra.

Table S2. Fraction of the probe inside ZIF-8 calculated from the CW EPR spectra.

**Sample**	**Fraction of loaded guest molecules, %**		**Sample**	**Fraction of loaded guest molecules, %**
**HEPES (pH=7.4)**	91		**MEM**	56
**HEPES (pH=7.8)**	94		**MEM + 10 mM MIM**	61
**PBS (pH=7.4)**	76		**MEM + 30 mM MIM**	61
**PBS (pH=7.8)**	88		**OPTIMEM**	27
**10% FBS**	94		**OPTIMEM + 10 mM MIM**	41
**10% FBS + 10 mM MIM**	93		**RPMI**	8
**199**	63		**RPMI + 10 mM MIM**	13
**199 + 10 mM MIM**	70		**α-MEM**	52
**DMEM**	30		**α-MEM + 10 mM MIM**	53
**DMEM + 10 mM MIM**	46			
**DMEM + 30 mM MIM**	58			
**IMDM**	15			
**IMDM + 10 mM MIM**	20			
**L-15**	11			
**L-15 + 10 mM MIM**	22			
**L-15 + 30 mM MIM**	36			
**Sample**	**Fraction of loaded guest molecules, %**		**Sample**	**Fraction of loaded guest molecules, %**
**Ala**	78		**Lys**	80
**Arg**	80		**Lys 1 mM**	87
**Asn**	59		**Lys 10 mM**	66
**Asn + 10 mM MIM**	77		**Met**	66
**Asp 2.5 mM**	65		**Met + 10 mM MIM**	76
**Cys**	17		**Phe**	47
**Cys + 10 mM MIM**	10		**Phe + 10 mM MIM**	63
**Cys 1 mM**	71		**Pro**	84
**Cys 10 mM**	1		**Ser**	61
**Cys 2.5 mM**	52		**Ser + 10 mM MIM**	85
**Glu**	73		**Ser 1 mM**	86

**Sample**	**Fraction of loaded guest molecules, %**		**Sample**	**Fraction of loaded guest molecules, %**
**Gly**	72		**Ser 10 mM**	51
**Gly + 10 mM MIM**	90		**Thr**	62
**His**	41		**Thr + 10 mM MIM**	73
**His + 10 mM MIM**	40		**Trp**	63
**His 1 mM**	86		**Trp + 10 mM MIM**	66
**His 10 mM**	4		**Tyr 1mM**	74
**His 2.5 mM**	66		**Val**	72
**Ile**	75		**Leu**	67

Parameters β, Ka, and fraction of spin probes escaped from ZIF-8 for studied amino acids.

Amino acid	Log(β)	pKa	Log(β)-2*pKa	Fraction of escaped guest molecules, %
Pro	9,69	10,34	-10,99	16,0
Arg	7,56	8,82	-10,08	20,4
Ala	8,56	9,5	-10,44	22,2
Ile	8,08	9,36	-10,64	25,3
Glu	8,54	9,39	-10,24	27,2
Gln	7,94	8,83	-9,72	27,8
Val	8,24	9,32	-10,4	27,9
Gly	9,01	9,38	-9,75	28,4
Met	6,93	8,91	-10,89	33,7
Trp	8,76	9,085	-9,41	36,7
Thr	8,14	8,71	-9,28	37,9
Ser	8,31	8,84	-9,37	39,1
His	11,68	8,92	-6,16	59,1
Cys	17,98	10,23	-2,48	83,5

We analyzed two processes: Zn^{2+}+2AA ↔ $Zn(AA)_2$ with constant β and HAA ↔ H^+ + AA^- with constant K_a, where AA is an amino acid and HAA indicates its protonated form. Equilibrium constants can be written as follows:

$$\beta = \frac{[ZnAA_2]}{[Zn^{2+}][AA^-]^2};\ K_a = \frac{[H^+][AA^-]}{[HAA]};$$

And concentration of deprotonated amino acids is

$$[AA^-] = \frac{K_a[HAA]}{[H^+]}.$$

Using this concentration in equation with β provides combination of β and K_a:

$$\beta = \frac{[ZnAA_2][H^+]^2}{[Zn^{2+}]K_a{}^2[HAA]^2};$$

$$\frac{\beta {K_a}^2 [HAA]^2}{{[H^+]}^2} = \frac{[ZnAA_2]}{[Zn^{2+}]}.$$

Since the whole process of ZIF-8 dissolution and zinc interaction with amino acids is complicated, we analyzed correlation between βK_a^2 and the amount of escaped radical.

Particle size distribution

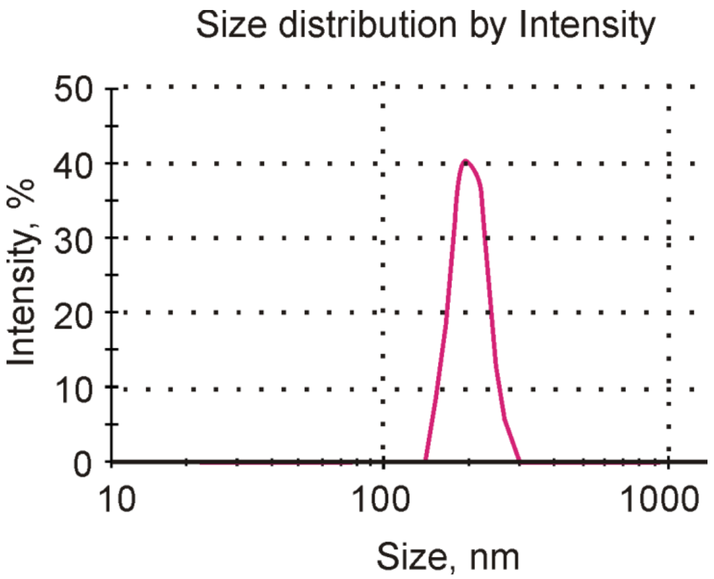

Figure S1. Particle size distribution curves of ZIF-8 nanoparticles in water measured by dynamic light scattering.

CW EPR spectra of R@ZIF-8 at pH=4.0 and free radical R in H_2O

Spectrum of fully dissociated R@ZIF-8 at pH=4 (citrate buffer) showed no differences compared to the spectrum of free radical in water.

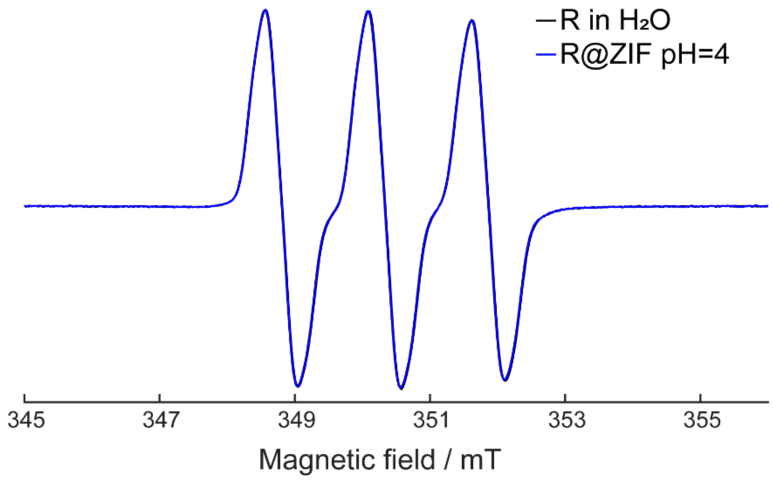

Figure S2. X-band room temperature CW EPR spectra of R@ZIF-8 at pH=4 and free radical R in water. Both spectra were normalized by double integral.

CW EPR spectra of initial R@ZIF-8 and R@ZIF-8 5-fold diluted with water

R@ZIF-8 dilution with water did not lead to MOF degradation: 99% of the spectrum corresponds to the loaded fraction.

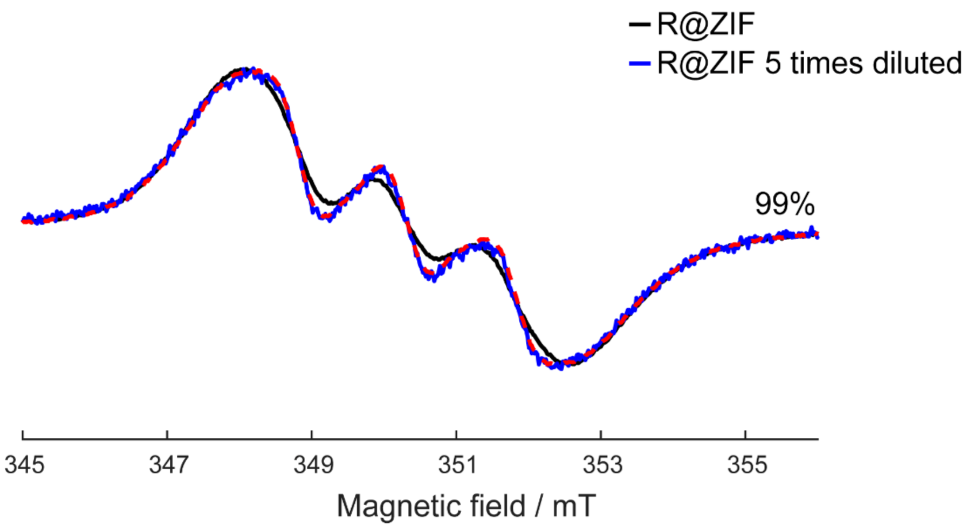

Figure S3. X-band room temperature CW EPR spectra of R@ZIF-8 and R@ZIF-8 diluted with water. Red dashed line shows spectrum simulation, the number on the right – the fraction of the probe inside ZIF-8. Spectra were normalized by double integral.

CW EPR of R@ZIF-8 in different media

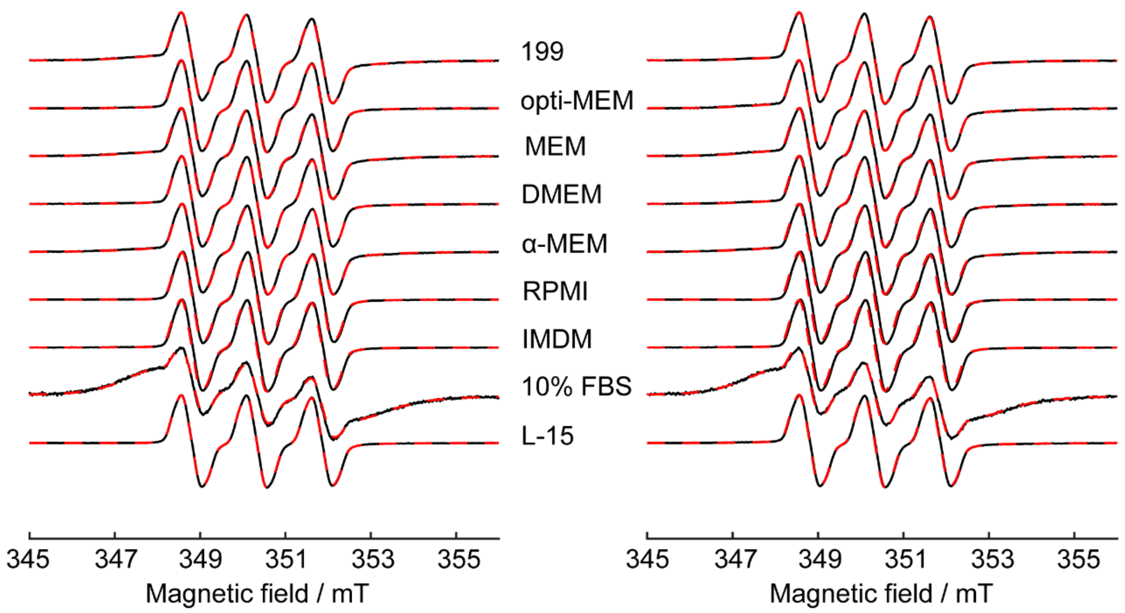

Figure S4. X-band room temperature CW EPR spectra of R@ZIF-8 after 5-fold dilution in culture media. On the left - spectra of samples without MIM, on the right - samples with

addition of 10mM MIM. Red dashed lines show simulations. All spectra were normalized by double integral.

Spectra of R@ZIF-8 in MEM at different concentrations

We diluted initial R@ZIF-8 with MEM 5-, 10- and 15-times v/v. The results demonstrated that the amount of escaped guest molecules increased with the amount of MEM added. The 10 and 15 times diluted sample spectrum had no component corresponding to the R@ZIF-8.

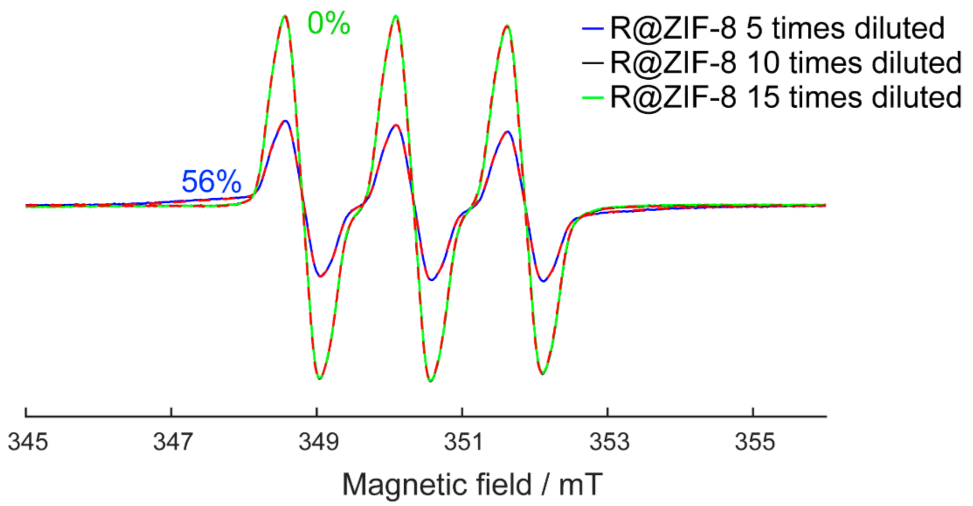

Figure S5. X-band room temperature CW EPR spectra of R@ZIF-8 and R@ZIF-8 diluted with MEM. Red dashed lines show spectra simulation, the numbers on the figure demonstrate fractions of loaded guest molecules, color code corresponds to the legend. All spectra were normalized by double integral.

EPR spectra of R@ZIF-8 with amino acids

All amino acids were dissolved in 5mM HEPES buffer, pH=7.4 at 5mM concentration except for Asp – 2.5mM Tyr – 1mM. Tyr and Asp were poorly soluble at this concentration.

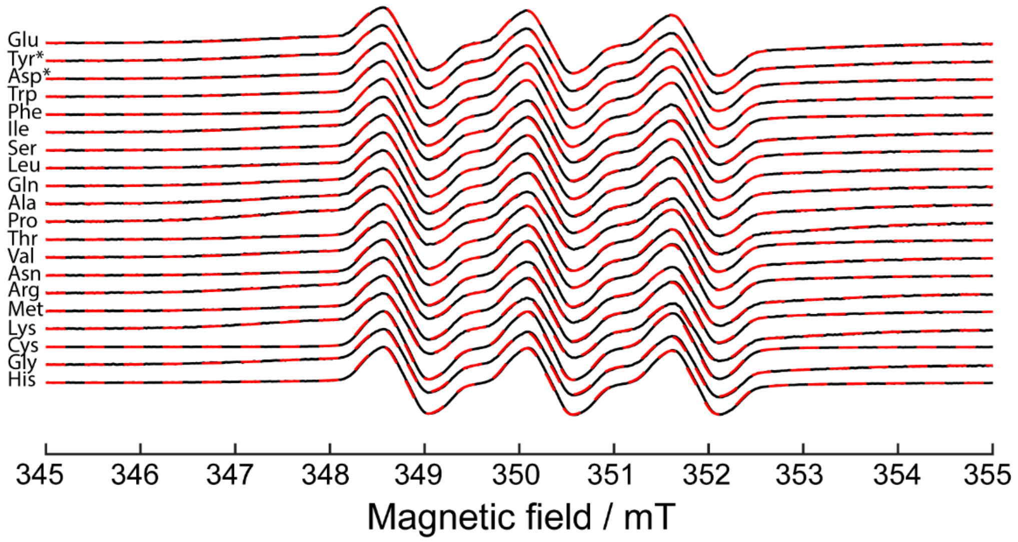

Figure S6. X-band room temperature CW EPR spectra of R@ZIF-8 after addition of amino acids with 5mM concentration (except Trp and Asp). Red dashed lines show simulations. All spectra were normalized by double integral.

EPR spectra at different concentrations of amino acids

All samples were made with a 5mM HEPES buffer at pH=7.4.

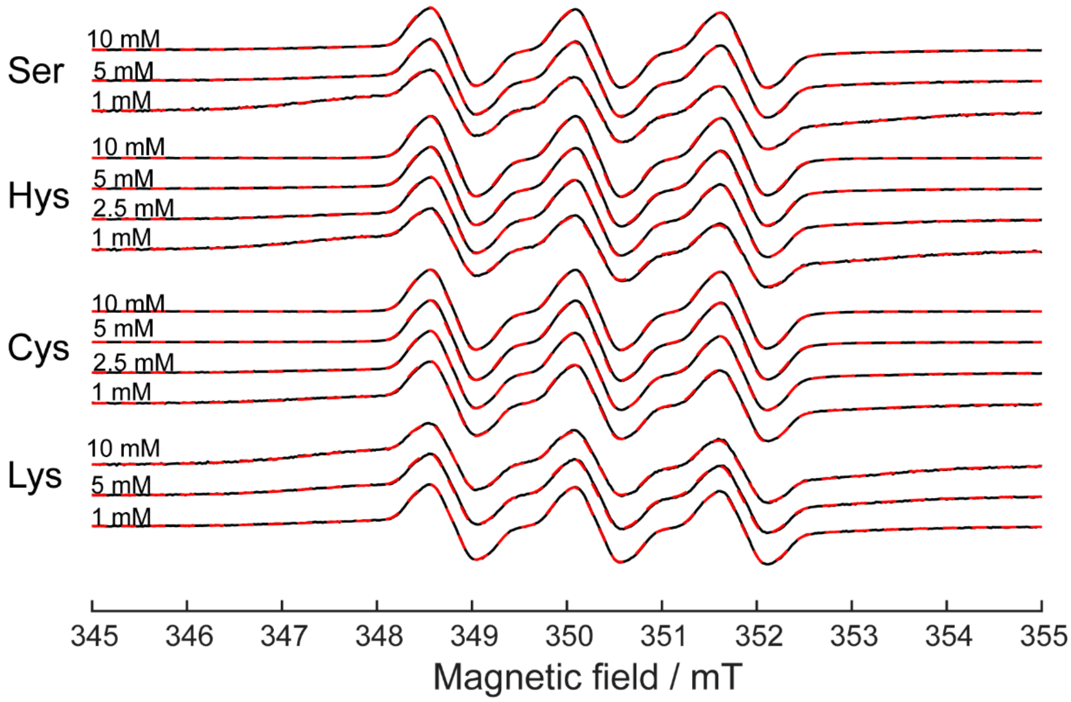

Figure S7. CW EPR spectra of R@ZIF-8 with amino acids addition at different concentrations. Red dashed lines show spectra simulation. All spectra were normalized by double integral.

EPR spectra of samples with 2-methylimidazole (MIM) addition.

We added 10mM MIM to all culture media and 9 amino acids to test whether MIM would stabilize nanoparticles. Moreover, MEM, DMEM and L-15 were tested with 30mM MIM. All solutions had pH=7.4. MIM addition resulted in increase of the broad spectrum component, therefore, the amount of dissolved nanoparticles decreased.

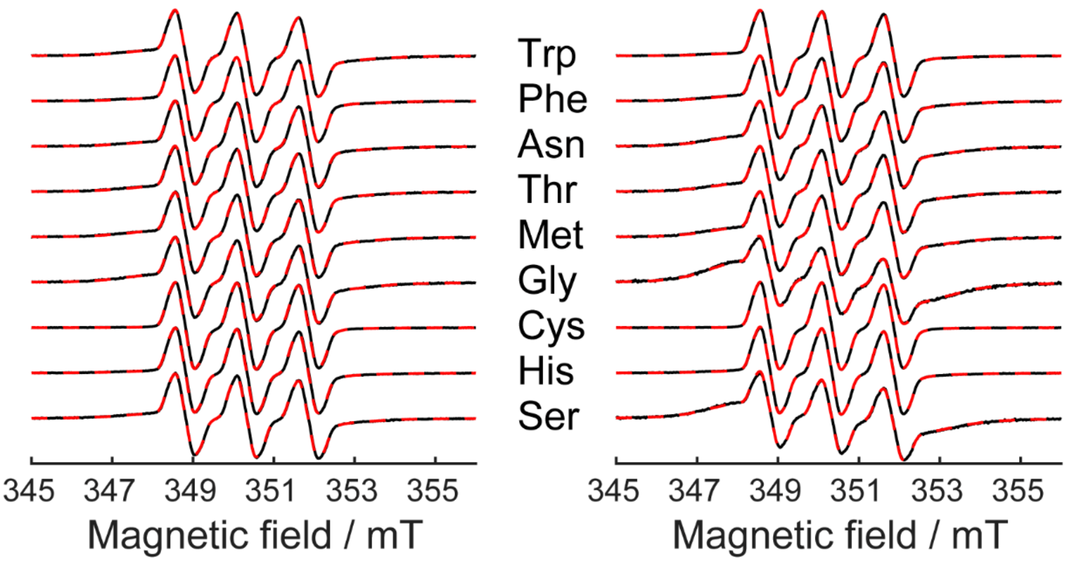

Figure S8. CW EPR spectra of R@ZIF-8 with addition of amino acids (5mM concentration). On the left - spectra of samples without MIM, on the right - samples with addition of 10mM MIM. Red dashed lines show spectra simulation. All spectra are normalized by double integral.

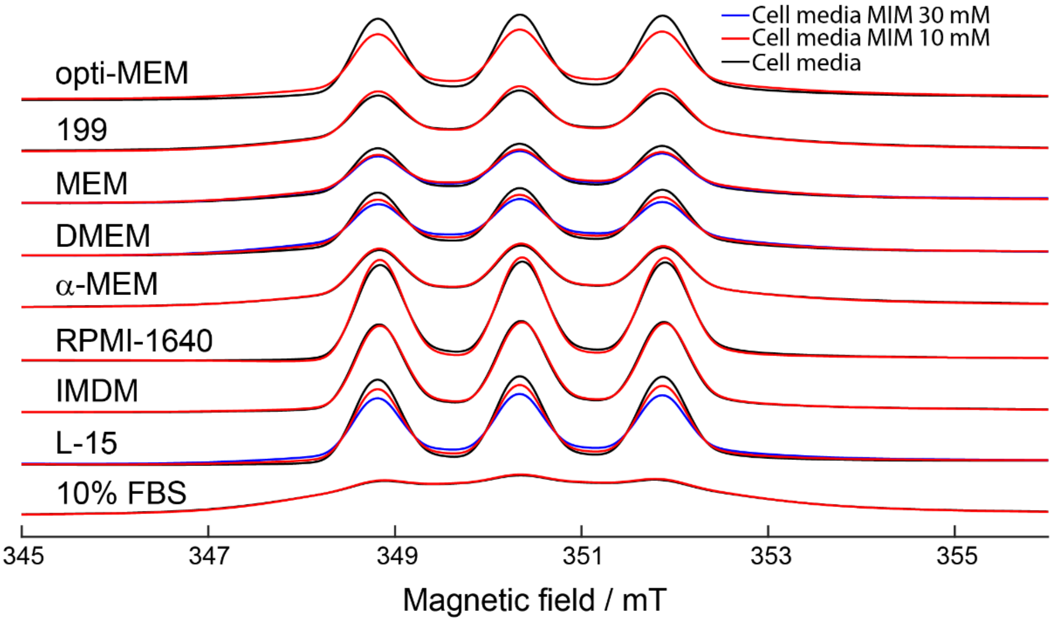

Figure S9. First integrals of CW EPR spectra of R@ZIF-8 after culture media addition, with and without MIM. All spectra are normalized by double integral.

Cytotoxic activity of MIM in A549 cells.

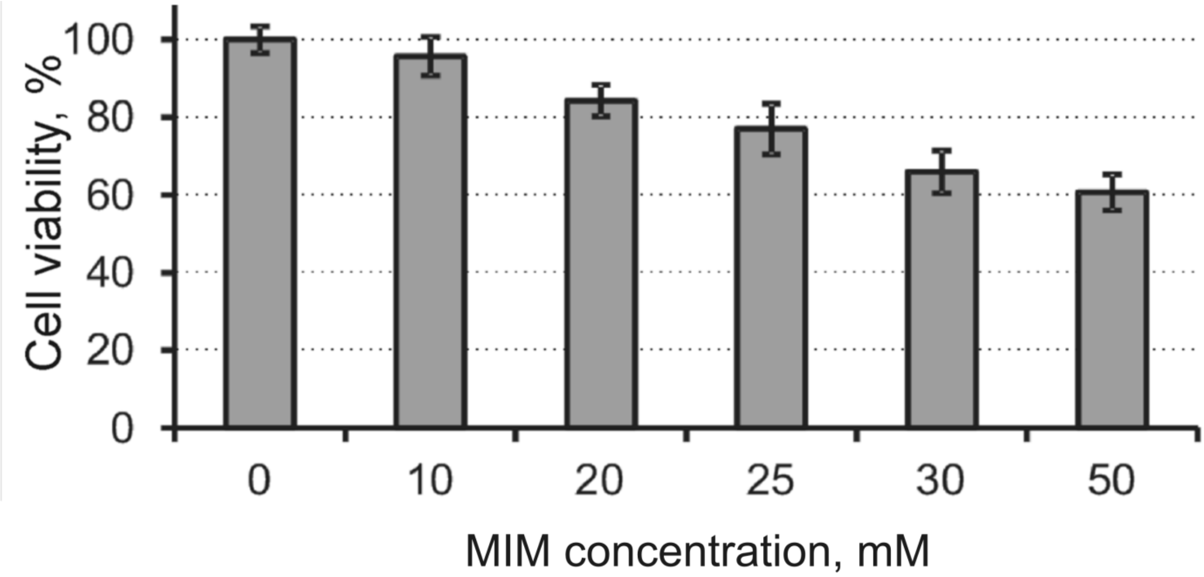

Figure S10. Cytotoxic activity of MIM in A549 cells. Cells were treated with different MIM concentration, and after 24 h of incubation, cells viability was analyzed by MTT assay.
